# High prevalence of undiagnosed hypertension among men in North Central Nigeria: Results from the Healthy Beginning Initiative

**DOI:** 10.1371/journal.pone.0242870

**Published:** 2020-11-30

**Authors:** Bolanle Feyisayo Banigbe, Ijeoma Uchenna Itanyi, Elizabeth Odilile Ofili, Amaka Grace Ogidi, Dina Patel, Echezona Edozie Ezeanolue

**Affiliations:** 1 APIN Public Health Initiatives, Abuja, Nigeria; 2 Department of Community Health Sciences, Boston University School of Public Health, Boston, Massachusetts, United States of America; 3 Center for Translation and Implementation Research, University of Nigeria Nsukka, Enugu, Nigeria; 4 Department of Community Medicine, University of Nigeria Nsukka, Enugu, Nigeria; 5 Clinical Research Center, Morehouse School of Medicine, Atlanta, Georgia, United States of America; 6 Department of Medicine, Morehouse School of Medicine, Atlanta, Georgia, United States of America; 7 Healthy Sunrise Foundation, Las Vegas, Nevada, United States of America; University of Mississippi Medical Center, UNITED STATES

## Abstract

**Background:**

The prevalence of hypertension in Nigeria is high and growing. The burden and risk factor distribution also vary by geographical zone. Information about prevalence, risk factors and disease status awareness are needed to guide evidence based public health response at the national and sub- national levels.

**Purpose:**

This paper describes the prevalence of hypertension and its correlates, as well as hypertension status awareness among men in North Central, Nigeria.

**Methods:**

A cross sectional survey was administered to male partners of pregnant women participating in the Healthy Beginning Initiative program from 2016–2018. Information on socio-demographic characteristics, risk factors, physical measurement and blood pressure readings were collected using a standardized protocol. Data was analyzed with simple and multiple logistic regression.

**Results:**

The 6,538 men had a median age of 31 years [IQR: 26–37]. The prevalence of hypertension was 23.3% (95% CI: 22.3%-24.4%), while 46.7% had prehypertension. The odds of hypertension was associated with increasing age (OR:1.02, CI:1.01–1.03), being overweight (aOR:1.5,CI:1.3–1.8), being obese (aOR:2.6,CI:2.0–3.3), living in an urban area (aOR:1.6,CI:1.2–2.1), and alcohol use in the 30 days prior (aOR:1.2,CI:1.1–1.4). Overall, 4.5% (297/6,528) of participants had ever been told they have hypertension. Among the 23.3% (1,527/6,528) with hypertension, 7.1% (109/1,527) were aware of their disease status. Men aged 41–50 years (aOR: 1.8, CI: 1.0–3.3), and > 50 years (aOR: 2.2, CI: 1.1–4.3), had higher odds disease status awareness. Living in an urban area was associated with lower odds (aOR: 0.2, CI: 0.03–0.7) of hypertension status awareness.

**Conclusion:**

This study showed that hypertension is already a significant public health burden in this population and that disease awareness level is very low. Alcohol use and obesity were associated with hypertension, highlighting some modifiable cardiovascular disease risk factors that are prevalent in the study population. Taken together, these findings can inform the design of interventions for primary and secondary cardiovascular disease prevention in Nigeria and similar settings.

## Introduction

Non-communicable diseases (NCDs) currently cause more deaths globally than all other causes combined [1, 2]. In 2016, they accounted for 71% of global mortality, translating to an estimated 41 million deaths [3]. Cardiovascular diseases, the most common NCDs, accounted for 44% of all NCD attributable deaths in 2016 [3] and more than 70% of premature NCD deaths, 75%-80% of which occurred in low and middle income countries (LMICs) [1, 2]. Uncontrolled hypertension is the singular most important risk factor for cardiovascular diseases including stroke, coronary heart disease, and chronic kidney disease [2, 4]. The world health organization (WHO) estimates that 22% of individuals aged 18 years and above, or about one billion people worldwide have hypertension, with Sub- Saharan Africa (SSA) having the highest prevalence at 27% [2, 3].

While infectious diseases and maternal and child health conditions are still the leading causes of death in Nigeria [5], the reported prevalence of hypertension has steadily increased from the 11.2% reported in a 1997 national survey [6] to about 28.9% [7] in 2013. Additionally, about 25% of hospital admissions among adults are due to complications of hypertension [8, 9] in a country with 209.6 million people in 2019 [10] and a life expectancy of 54 years in 2018 [11]. Although Nigeria has not conducted a recent national NCD survey at the time of this study, several small and medium sized studies have reported prevalence estimates from different parts of the country. The reported prevalence ranges from 30.6% [12] in hospital settings to about 30% [13–16] in communities and 48% [17] among formal sector workers depending on study size, participant age and location.

The prevalence and distribution of modifiable risk factors for hypertension in Nigeria also vary by geo-political zone [13–16, 18].However, Northern Nigeria in general and the North Central geo-political zone in particular is not well represented in existing studies. In a 2015 meta-analysis by Adeloye et al, 85% of the studies were conducted in the southern parts of Nigeria [7]. Given the socio-economic, demographic and cultural diversity of the Nigerian population, information about prevalence and risk factor distribution in one zone may not be completely generalizable to other geo-political zones [15].

The goal of this paper was to determine the prevalence of hypertension and its correlates, as well as hypertension status awareness among a population of men in Benue state, North Central Nigeria.

## Methods

### Study setting, design, and participants

We conducted a cross-sectional study among a cohort of pregnant women and their male partners (expectant couples), who participated in the Healthy Beginning Initiative (HBI) program in Benue State, Nigeria.

Benue State is located in the North Central geo-political zone, it has 23 local government areas (LGAs) and an estimated population of five million [19], of which about 70% are farmers. A total of eighty churches in 80 communities across twelve LGAs in Benue State participated in this program, from June, 2016 to October 2018. Benue State has the second highest prevalence of HIV in Nigeria, at 4.9% [20].

The HBI program provides an integrated, feasible and culturally adaptive platform for HIV/Hepatitis B screening, linkage and follow up of pregnant women, aiming to identify, treat and retain women in care throughout their pregnancy. Details of the HBI program have been described elsewhere [21, 22]. In brief, HBI consists of three components that are all congregation-based: 1) **Prayer sessions**, in which every Sunday at the end of the church service, the priest or pastor announced for pregnant women and their male partners to come out for prayers. He prayed for a healthy pregnancy, safe delivery and encouraged pregnant women to seek antenatal care at a health facility. 2) **Baby showers** were organized as a reception and health fair in churches, during which health education on early antenatal care, importance of the integrated screening tests for pregnant women, good nutrition, skilled birth attendance, and immunizations were provided. This provided an opportunity for onsite integrated laboratory testing for HIV, Hepatitis B virus and sickle cell genotype to be conducted for pregnant women and their male partners. Male partners received a “mama pack” to present to their female partners as an expression of love and support during the pregnancy. 3) **Baby receptions** were organized six to eight weeks post-delivery, for women who participated in the baby shower, where women also completed a post-delivery questionnaire to ascertain place of delivery and pregnancy outcome. Enhanced nutrition and immunization education were provided. This also offered an opportunity for post-delivery linkage to care for women as needed.

### Questionnaire administration

Trained program assistants who had a minimum of a bachelor’s degree collected the data for this study using a pretested semi-structured questionnaire. After participants arrived for the baby showers, they prayed and received general health education allowing time for participants to settle down and relax. Each pregnant woman and her male partner were then invited into a private location where the questionnaires were administered, and physical measurements taken including weight and blood pressure. There were separate male and female questionnaires. Only data from the male questionnaires will be presented in this paper. Data were collected on sociodemographic characteristics, medical history, and lifestyle habits of the participants.

Sociodemographic data included age, sex, marital status, highest level of education, occupation, monthly income, languages spoken, distance to health facility, and number of people living in the household. Past history of hypertension and/or diabetes diagnosis were assessed via self-reporting. Data was also collected on lifestyle habits including tobacco, alcohol and other substance use.

### Physical measurements

The physical measurements taken from the participants were blood pressure, weight and height. Each participant had his weight measured once, using calibrated analog bathroom weighing scales. The weighing scale was placed on a flat and firm surface. Participants were asked to remove any heavy clothing or objects and stand at the centre of the scale. Trained program assistants read the measured weights to the nearest 1 kilogram (kg) and recorded them in a designated form.

Height was measured once for each participant using a customized metre rule. Participants were asked to stand on a flat surface with feet flat and together, and against a wall, and ensuring that participant looked straight ahead with the line of sight parallel to the floor. With the head, shoulders, buttocks, and heels touching the wall, a small flat object (eg. ruler) was used to form a right angle between the wall and the crown of the head. A light mark was made on the wall where the object touched the wall, and the height was recorded to the nearest 0.01 metre.

Blood pressure (BP) was measured by trained program assistants using a digital sphygmomanometer by Omron Healthcare Inc, USA. Measurements were taken for each participant after an initial resting period, with the participant seated with feet flat on the floor, a properly sized cuff was wrapped round the right arm, with the lower edge one inch above the antecubital fossa. The cuff was inflated by turning on the device and the displayed systolic and diastolic blood pressures were recorded. Each participant had his blood pressure measured once.

### Measurement of NCD risk factors

Body mass index was calculated as weight (kg) divided by the square of height (m), and classified as underweight if <18.5 kg/m^2^, normal if 18.5–24.9 kg/m^2^, overweight if 25–29.9 kg/m^2^, and obese if > 30 kg/m^2^ according to WHO recommendations [23]. Current alcohol consumption was defined as having an alcoholic drink within the last one month, while current tobacco consumption was defined as tobacco use within the last 30 days. We classified blood pressure according to the JNC-7 guidelines as follows: normal (systolic BP <120mmHg and diastolic BP< 80mmHg), pre-hypertension (SBP; 120–139 mmHg and DBP 80–89 mmHg), stage 1 hypertension (SBP: 140–159 mmHg and/or DBP: 90–99 mmHg), stage 2 hypertension (SBP: ≥ 160mmHg or DBP ≥ 100mmHg) [24]. All participants with self-reported history of hypertension were categorized into the hypertension group if their blood pressure was above 140/90 mmHg, otherwise they were categorized as not hypertensive or pre-hypertensive as appropriate according to the JNC-7 criteria.

### Hypertension awareness

We assessed awareness of hypertension status by asking each participant, “has any medical professional (nurse or doctor) ever told you that you had hypertension before this baby shower?”. Participants were categorized as being aware of their disease status if they answered “Yes” to this question and had a blood pressure reading above 140/90mmHg.

### Data analysis

Continuous variables were summarized using their mean and standard deviation or median and interquartile range as appropriate, categorical variables were expressed as percentages. We determined the prevalence of hypertension and disease status awareness in the study population. Bivariate analysis was done using chi-square tests of association or Fishers exact test for categorical independent variables, and t-tests or Kruskal-Wallis tests of association for continuous variables as appropriate. We built bivariate and multiple logistic regression models to assess the factors associated with hypertension and hypertension status awareness separately. Covariates demonstrating marginally significant bivariate associations (p< = 0.1) were included in multiple regression models. We used the most parsimonious models by removing covariates that did not exhibit statistically significant association with the outcome at a p-value of <0.05 via a backwards elimination procedure. The least significant variable was removed in each iteration and this was repeated until all variables in the model had a P-value of < 0.05. Data was analyzed using R statistical software (The R Foundation for Statistical Computing, 2019).

### Ethical considerations

This study was approved by the Health Research Ethics Committee of the University of Nigeria Teaching Hospital, Enugu, Nigeria. Although consent was not needed to participate in the baby showers, written informed consent was obtained from the participants for questionnaire data collection and physical measurements.

## Results

A total of 6,766 men participated in the HBI all of whom completed the questionnaires and had their physical measurements taken. Two hundred and twenty-eight records (3.4%) were excluded from the analysis due to implausible blood pressure values, and age less than 18 years; here we report on the findings on the 6,538 men included in the final analysis. The median age in our population was 31 years (IQR: 26–37 years), and a majority of the participants (49.8%) were in the 18–30 age category (Table 1). Overall, about 45% of the participants completed secondary education, most (82.5%) earned less than 20,000 Naira or approximately US$56 per month (US$1 = 360 Naira in 2016), and a majority (77.8%) lived in rural areas. The median BMI was 23.1 (IQR: 21.2–25.3), and 25.4% of the participants were overweight or obese. Forty eight percent (48.2%) reported current alcohol consumption, and 25.1% of the men reported using tobacco in the past 30 days. Among those who reported using tobacco, 81% used it daily. About four percent (3.9%) of the men reported using other substances apart from tobacco, the three most commonly used substances were snuff (68%), tramadol (9.9%), and Indian hemp (8.3%). The mean systolic BP was 126.9 mmHg (SD: 16.7mmHg), and mean diastolic BP was 76.7 mmHg (SD: 12.2mmHg). About five percent (4.5%) and 2.3% of men in this study reported ever being told by a doctor that they had hypertension and diabetes, respectively.

**Table 1:** Characteristics of study participants in Benue, Nigeria.

Variable	Total	Hypertension +ve (N = 1,527)	Hypertension -ve (N = 5,011)	p-value
Age (years), Median (IQR)	31 (26–37)	32 (27–40)	30(26–37)	<0.001*
Age Categories, N (%)				
18–30 years	3256 (49.8%)	682 (44.7%)	2574 (51.4%)	<0.001^#^
31–40 years	2223 (34%)	509 (33.3%)	1714 (34.2%)	
41–50 years	732 (11.2%)	210 (13.8%)	522 (10.4%)	
>50 years	327 (5%)	126 (8.3%)	201 (4%)	
BMI Overall, Median (IQR)	23.1 (21.2–25.3)	23.8(21.6–26.2)	22.9 (21–24.8)	<0.001*
BMI Categories, N (%)				
Underweight	315 (4.8%)	51 (3.3%)	264 (5.3%)	<0.001^#^
Normal	4,665 (69.2%)	949 (62.1%)	3616(72.2%)	
Overweight	1,366 (20.9%)	403 (26.4%)	963 (19.2%)	
Obese	292 (4.5%)	124(8.3%)	168(3%)	
Education, N (%)				
No formal education	369 (5.6%)	84 (5.5%)	285 (5.7%)	0.004^#^
Completed Primary education	2,524 (36.6%)	573 (37.5%)	1951(38.9%)	
Completed Secondary Education	2,935 (44.9%)	666(43.6%)	2269(45.3%)	
Completed Tertiary Education	708 (10.8%)	204 (13.4%)	504(10.1%)	
Monthly Income, n (%)				<0.001^#^
0–20,000	5,393 (82.5%)	1202(78.8%)	4191(83.7%)	
20,001–50,000	794 (12.2%)	213 (14%)	581(11.6%)	
50,001–100,000	261 (3.9%)	82 (5.4%)	179 (3.6%)	
>100,000	85 (1.3%)	29 (1.9%)	56 (1.1%)	
Alcohol use in the 30 days prior, N (%)	3,151 (48.2%)	801 (52.5%)	2350 (46.9%)	0.0002^#^
Tobacco Use in 30 days prior, N (%)	1,643 (25.1%)	371 (24.3%)	1272 (25.4%)	0.42^#^
Other Substance Use, N (%)	252 (3.9%)	57 (3.7%)	195 (3.9%)	0.8
Location, N (%)				<0.001^#^
Rural	5,087(77.8%)	1178 (77.1%)	3909 (78%)	
Semi -urban	1,204 (18.4%)	263 (17.2%)	941 (18.8%)	
Urban	247 (3.8%)	86 (5.6%)	161 (3.2%)	
Systolic blood pressure, Mean (SD)	126.9 (16.7)	148.2 (15.1)	120.5 (10.7)	<0.001^&^
Diastolic blood pressure, Mean (SD)	76.7 (12.2)	89.5 (13.2)	72.8 (8.8)	<0.001^&^
Self-reported history of Diabetes, N (%)	153 (2.3%)	44 (2.9%)	109 (2.2%)	0.13^#^
Self-reported history of hypertension, N (%)	297 (4.5%)	109 (7.1%)	188 (3.8%)	<0.001

* Kruskal- Wallis test,

^#^ Chi-square Test,

^&^ t-test.

About a quarter (23.3%) [95% CI: 22.3%-24.4%] of the men in this study had hypertension, while 46.7% had pre-hypertension as shown in Table 2. Only a third of the participants had normal blood pressure readings while about 6% had stage 2 hypertension. The crude prevalence of hypertension increased with increasing age in this population.

**Table 2:** Prevalence of hypertension and pre-hypertension among men in Benue, Nigeria.

Variable	Total
Hypertension N (%)	1527 (23.3%)
Hypertension Stage, N (%)	
Normal	1,959 (30%)
Pre-hypertension	3,052 (46.7%)
Stage 1	1,139 (17.4%)
Stage 2	388 (5.9%)
Age Category of participants with hypertension	
18–30 years	682 (20.9%)
31–40 years	509 (22.9%)
41–50 years	210 (28.7%)
>50 years	126 (38.5%)

In simple logistic regression models, (Table 3), older age was associated with hypertension, compared to men less than 30 years old; men in the 5^th^, 6^th^, and 7^th^ decades of life had higher odds of hypertension with odds ratio of 1.5 [95% CI: 1.3–1.8], and 2.4 [95% CI: 1.9–3.0] respectively. High body mass index was also associated with having hypertension, the odds of hypertension increased by 1.09 for every unit increase in BMI [95% CI: 1.07–1.1]. The odds of hypertension was 1.6 times higher [95% CI: 1.4–1.8] in overweight subjects and 2.8 times higher in obese subjects [95% CI: 2.2–3.6] compared to those with normal BMI. Alcohol consumption in the last 30 days was associated with higher odds of being hypertensive [OR: 1.3, 95%CI: 1.1–1.4], so was completing tertiary education [OR: 1.4, 95% CI: 1.1–1.7]. Income was also associated with increased odds of being hypertensive, with the odds increasing as reported income increased; compared to subjects in the lowest income category (less than 20,000 Naira per month), those in the highest income category had almost 2 times the odds of being hypertensive [OR: 1.8, 95% CI: 1.1–2.8]. Men who reported living in urban areas had higher odds of being hypertensive [OR: 1.8, 95%CI: 1.4–2.3] compared to those living in rural areas. There was no significant association between subjects being told they had diabetes and the odds of being hypertensive in this population [OR: 1.3, 95%CI: 0.9–1.9]. There was also no significant relationship between tobacco use in the last 30 days and being hypertensive. [OR: 0.94, 95%CI: 0.83–1.1].

**Table 3:** Association of selected risk factors with prevalence of hypertension among men in Benue, Nigeria.

		Simple Logistic Regression		Multiple Logistic Regression	
Variable		Unadjusted Odds Ratio (95% Confidence Interval)	p-value	Adjusted Odds Ratio (95% Confidence Interval)	p-value
Age Categories	18–30 years	1		1	
	31–40 years	1.1 (0.9–1.3)	0.09	1.03 (0.9–1.2)	0.7
	41–50 years	1.5 (1.3–1.8)	<0.01	1.4 (1.2–1.7)	0.0004
	>50 years	2.4 (1.9–3.0)	<0.01	2.4 (1.8–3.0)	<0.01
	Age in years	1.02 (1.01–1.03)	<0.01		
	Overall BMI	1.09 (1.07–1.1)	<0.001		
BMI Categories	Normal	1		1	
	Underweight	0.7 (0.5–0.99)	0.05	0.7 (0.5–0.9)	0.01
	Overweight	1.6 (1.4–1.8)	0.001	1.5 (1.3–1.8)	0.001
	Obese	2.8 (2.2–3.6)	0.001	2.6 (2.0–3.3)	0.001
Education	Completed Primary education	1		1	
	No formal education	1.0 (0.8–1.3)	0.9		
	Completed Secondary Education	0.99 (0.9–1.1)	0.9		
	Completed Tertiary Education	1.4 (1.1–1.7)	0.0008		
Monthly Income	0–20,000	1		1	
	20,001–50,000	1.3 (1.1–1.5)	0.005		
	50,001–100,000	1.6 (1.2–2.1)	0.0006		
	>100,001	1.8 (1.1–2.8)	0.01		
Location	Rural	1		1	
	Semi -urban	0.9 (0.8–1.1)	0.33	0.9 (0.8–1.1)	0.2
	Urban	1.8 (1.4–2.3)	<0.001	1.6 (1.2–2.1)	0.001
Alcohol Use		1.3 (1.1–1.4)	0.001	1.2 (1.1–1.4)	0.001
Current tobacco use		1.0 (0.8–1.1)	0.4		
Self-reported history of diabetes		1.3 (0.9–1.9)	0.1		

In the final multiple logistic regression model, being above 40 years, being overweight [aOR: 1.5, 95%CI: 1.3–1.8], being obese [aOR: 2.6, 95%CI: 2.0–3.3], living in an urban area [aOR: 1.6, 95%CI: 1.2–2.1], and alcohol use in the 30 days prior [aOR: 1.2, 95%CI: 1.1–1.4] were significantly associated with increased odds of hypertension in this population.

Two hundred and ninety-seven (297) or 4.5% of men in the study responded “Yes” to the question “have you ever been told that you have hypertension?” Of these men, 109 recorded raised blood pressure, while 188 did not have raised blood pressure at the time of taking the measurements. We found that 93% of participants with hypertension were unaware of their diagnosis. Awareness of hypertension diagnosis was highest among participants living in rural areas (79.8%) and lowest among urban (1.8%) participants (Table 4). Hypertension status awareness was 4.8%, 5.5%, 12.9%, and 16.7% among the 18–30 years, 31–40 years, 41–50 years, and > 50 years age categories respectively.

**Table 4:** Socio-demographic characteristics of participants who were aware of their hypertension diagnosis.

Variables	Aware (N = 109)	Not Aware (N = 1,418)	P-value
Age Categories, N (%)			
18–30 years	33 (4.8%)	649 (95.2%)	<0.001^#^
31–40 years	28 (5.5%)	481 (94.5%)	
41–50 years	27 (12.9%)	183 (87.1%)	
>50 years	21 (16.7%)	105 (83.3%)	
Education, N (%)			0.0002^#^
No formal education	10 (9.2%)	74 (5.2%)	
Completed Primary education	27 (24.8%)	546 (38.5%)	
Completed Secondary Education	45 (41.3%)	621 (43.8%)	
Completed Tertiary Education	27 (24.8%)	177 (12.5%)	
Monthly Income, N (%)			<0.001^^^
0–20,000	68 (62.4%)	1134 (80%)	
20,001–50,000	24 (22%)	189 (13.3%)	
50,001–100,000	15 (13.8%)	67 (4.7%)	
>100,000	2 (1.8%)	27 (1.9%)	
Location, N (%)			0.19^
Rural	87 (79.8%)	1091 (76.9%)	
Semi -urban	20(18.3%)	243 (171%)	
Urban	2 (1.8%)	84 (5.9%)	

^ Fishers exact test,

^#^ Chi-square test.

In simple logistic regression models, (Table 5), older age was associated with hypertension status awareness, compared to men less than 30 years old; men aged 41–50 years had 2.9 [95% CI: 1.7–4.9] times the odd of being aware of their status, and men > 50 years had 3.9 [95% CI: 2.2–7.0] times the odd of status awareness. Having no formal education [OR: 2.7, 95%CI: 1.2–5.7], completing tertiary education [OR: 3.1,95% CI: 1.8–5.4], and monthly income between 20,000 and 100,000 Naira were associated with higher odds of status awareness. In the final multiple logistic regression model (Table 5), men aged 41–50 years [aOR: 1.8, 95%CI: 1.0–3.3], and >50years [aOR: 2.2, 95%CI: 1.1–4.4] had higher odds of being aware of their hypertension diagnosis. Men who earned 20,001–50,000 Naira [aOR: 2.5, 95%CI: 1.4–4.3] and 50, 0001–100,000 Naira per month [aOR: 2.7, 95%CI: 1.2–5.6] respectively also had higher odds of hypertension status awareness. Living in an urban area was associated with lower odds [aOR: 0.2, 95% CI: 0.03–0.7] of hypertension status awareness.

**Table 5:** Factors associated with hypertension status awareness among men in Benue, Nigeria.

Variable		Unadjusted Odds Ratio (95% Confidence Interval)	p-value	Adjusted Odds Ratio (95% Confidence Interval)	p-value
Age Categories	18–30 years	1		1	
	31–40 years	1.1 (0.7–1.9)	0.6	0.8(0.4–1.4)	0.3
	41–50 years	2.9(1.7–4.9)	<0.001	1.8 (1.0–3.3)	0.04
	>50 years	3.9 (2.2–7.0)	<0.001	2.2 (1.1–4.3)	0.03
Education	Completed Primary education	1		1	
	No formal education	2.7 (1.2–5.7)	0.01	2.2 (0.9–4.9)	0.08
	Completed Secondary Education	1.5 (0.9–2.4)	0.1	1.3 (0.8–2.3)	0.3
	Completed Tertiary Education	3.1 (1.8–5.4)	<0.001	2.5 (1.3–4.7)	0.007
Monthly Income	0–20,000	1		1	
	20,001–50,000	2.1 (1.3–3.4)	0.003	2.5 (1.4–4.3)	0.001
	50,001–100,000	3.7 (2.0–6.7)	<0.001	2.7 (1.2–5.6)	0.01
	>100,001	1.2 (0.2–4.2)	0.8	0.7 (0.1–3.0)	0.7
Location	Rural	1		1	
	Semi–urban	1.03 (0.6–1.7)	0.9	1.03(0.6–1.8)	0.9
	Urban	0.3 (0.04–0.9)	0.09	0.2 (0.03–0.7)	0.03
Alcohol Use		1.3 (0.9–1.9)	0.3		
Current tobacco use		0.7 (0.4–1.1)	0.2		

## Discussion

This study examined the prevalence and correlates of hypertension, as well as hypertension status awareness among a population of men in North Central Nigeria. About one in four of the participants had hypertension. Older men, those who were overweight or obese, men reporting current alcohol use, and those residing in an urban area had higher odds of hypertension. More than 40% of the men in our study had pre-hypertension, while 20.9% and 4.5% were overweight and obese, respectively. Perhaps more important is that 93% of the participants with hypertension in this study were not aware of their disease status. Age, income, completing tertiary education and living in a rural area were associated with higher odds of hypertension status awareness.

Previous community-based studies in Nigeria have reported higher hypertension prevalence, between 34.9% and 46.3% [25–28] among men, however, our study population was younger than those in many of these studies, which could have accounted for the difference in prevalence. Our finding is however similar to the pooled prevalence among Nigerian men of 29.5% reported in a 2015 meta-analysis [7], and a prevalence of 26.1% in another meta-analysis estimating the burden of hypertension in the Niger- delta region of Nigeria [29]. The odds of hypertension increased with increasing age in our study, aligning with the already established relationship between hypertension and increasing age in Nigeria [7, 25–30], and globally [31].Previous diagnosis of diabetes was self-reported therefore we could have underestimated the proportion of men with history of diabetes because of the possibility of undiagnosed diabetes. This may have contributed to the lack of association between hypertension and history of diabetes in this study despite established evidence of such association [32].

Notably, the hypertension status awareness in the present study is much lower than earlier Nigerian studies which have reported awareness levels ranging between 13.9% and 40.3% among men [25, 33–35],although none has been reported for this study location. In their meta-analysis, Adeloye et al also estimated that 17% of hypertensives in Nigeria were aware of their diagnosis [7]. Our finding is similar to the 5%-10% awareness rate reported in some regions of Tajikistan [36], and the 7.4% awareness rate reported among the urban poor in Ghana [37], although much lower than the 17.5% reported in a pooled study of 44 LMICs in four regions of the world [38], the 44.7% in China [39], and the 63.8% in the United States [40]. Globally, undiagnosed hypertension remains a problem [39–43], with some patients “hiding in plain sight” even in high income countries like the United States [40]. This is most likely because hypertension is asymptomatic until it causes end organ damage. The low awareness rate is worse in LMICs with poor access to primary care [41]. Regardless of the exact proportion, the available evidence suggests that low hypertension status awareness, and inadequate diagnosis of hypertension are important drivers of cardiovascular disease morbidity and mortality [27]. Our paper contributes to the evidence of the need to scale up feasible, culturally acceptable, and sustainable alternative strategies for hypertension screening such as community-based screening to increase hypertension status awareness globally.

Hypertension status awareness is critical because although cheap and effective treatment exists for hypertension, status awareness is one of the earliest steps in the hypertension care cascade [44]. Being unaware of one’s hypertension status therefore means individuals are not able to receive the treatment they need until they develop advanced or complicated disease, which is more difficult and more expensive to treat. This is especially important for a country like Nigeria, where close to 25% of adult medical admissions are due to complications of hypertension. Studies reviewing adult medical inpatient admissions consistently place hypertension and its complications among the top five causes of hospital admission and mortality [45–48]. Additionally, there is evidence that many hypertensive patients already have end organ damage at presentation for initial care in Nigeria [49, 50]. These findings suggest that many Nigerians with hypertension may be receiving their diagnosis late, and suffering avoidable morbidity and mortality as a result.

The effect of age, rural-urban location, income and education on hypertension status awareness are mixed in many studies [27, 44]. Kayima et al reported that older age, being educated, having health insurance coverage and living in an urban area were associated with higher rates of awareness across Africa [44]. A rather surprising finding in our study is that rural and semi-urban dwellers had higher odds of being aware of their diagnosis than those who lived in urban areas. This is different than findings from other studies [27, 41, 44], and what is expected considering that urban areas tend to have more health facilities than rural areas. A possible explanation could be that rural areas tend to benefit more from free medical missions and outreaches which may partially ameliorate the financial barrier to healthcare access that is common across Nigeria [51, 52], and could have provided rural dwellers in this study with opportunities to have their blood pressure measured. The small proportion of urban dwellers in this study population may also have contributed to this finding. Overall, the finding of low awareness of personal hypertension status points to the need for innovative yet sustainable strategies to increase hypertension awareness across all geographical locations, including among populations with low socio-economic status in middle and high income countries [42].

The mixed findings on correlates of disease status awareness suggests that perhaps there were unmeasured confounders in our study population and points to the need for more research to understand the drivers of disease status awareness.

The finding that 46.7% of men had prehypertension is similar to findings from earlier studies in Nigeria which reported prehypertension rates ranging between 42.8%- 48.4% among men [53–55], but lower than the 59.2% reported by Isezuo et al [35] in Northern Nigeria. While prehypertension is not a disease in itself [24], it confers a 2–3 fold higher risk of incident hypertension on individuals, and is independently associated with heart disease [24, 56, 57]. It has a reported 4%-9% annual risk of progression to hypertension [57], depending on the presence of other cardiovascular disease risk factors such as diabetes, obesity and hypercholesterolemia [57, 56]. Additionally, evidence from the United States suggests that transition from prehypertension to hypertension is faster among black people [24, 57]. While pharmacological management is not recommended for prehypertension in the absence of other risk factors, identifying patients in this category presents an opportunity for early initiation of low cost lifestyle interventions for cardiovascular disease risk reduction [35, 56–58]. Implementation Science approaches can inform the best strategies for integrating such risk reduction interventions into the current service delivery system in Nigeria.

About a quarter of the men in this study were overweight or obese, and this was significantly associated with the odds of hypertension, with the odds increasing with every unit increase in BMI. Our study corroborates earlier studies that have established a firm relationship between being hypertensive and raised BMI [26, 28, 29, 33, 55]. In a recent study that analyzed data from 13 African countries, obese Africans were two to eight times as likely to be hypertensive compared to those with normal BMI, the odds of which increased with age [59]. Given this established association between obesity and hypertension, as well as its high prevalence, this is an important modifiable risk factor for public health interventions in Nigeria. Harmful alcohol use is also associated with the increased risk of hypertension [60, 61], and almost half of the men in this population reported using alcohol in the last 30 days. Even though our study did not assess intensity of use, current alcohol use was associated with higher odds of hypertension in the adjusted regression model. Our findings agree with previous studies that found alcohol intake and/or binge drinking was associated with hypertension among Nigerians [50, 62, 63]. The high prevalence of alcohol use in this study population, which is similar to what obtains in the general population [64] suggests that alcohol use is another modifiable risk factor that can be targeted for both primary and secondary prevention of hypertension in Nigeria.

One of the strengths of this study is that, to our knowledge, this is the first in Nigeria to focus on men. This is especially important since men tend to constitute a smaller proportion of participants in past community-based studies of hypertension prevalence in Nigeria, and some studies have identified male gender as a risk factor for hypertension. While this was not a population-based study, religion plays a very important role in social life in Nigeria with close to 90% of the population attending a religious service once a week [65], therefore our likelihood of capturing the population in the study communities was high. Additionally, pregnant women received presents from their partners during the baby showers as a show of support, leading to high levels of participation among men [22]. Our study however has limitations; it is a study among spouses of pregnant women who were generally younger, so the prevalence of hypertension may be higher in the community. An additional limitation of our study is that blood pressure measurements were taken only once, and we did not ask if any of the participants were currently taking anti-hypertensives; this could have led to underestimation of the prevalence in this study if some of the men had their hypertension controlled on medications. The very low proportion (4.5%) of participants who reported that they have been told they have hypertension makes this unlikely. Furthermore, due to the cross-sectional nature of the present study and the subjective nature of some of the socio-demographic variables, we are unable to determine causality between socio-demographic factors and the prevalence or awareness of hypertension. Finally, the authors acknowledge that use of odds ratio may have overestimated the association between hypertension as well as hypertension awareness and independent variables compared to prevalence ratio.

## Conclusions

Our current study’s finding supports previous findings that undiagnosed hypertension remains a major problem globally. It demonstrates that in resource-limited settings where access to health facilities are constrained, community screening for hypertension is feasible, acceptable, and complementary to health facility-based screening especially if integrated with existing community programs.

## Supporting information

S1 Dataset (XLSX)

## References

[1] Benziger CP, Roth GA, Moran AE. The Global Burden of Disease Study and the Preventable Burden of NCD. Glob Heart 2016;11:393–7. 10.1016/j.gheart.2016.10.024 27938824

[2] World Health Organization (WHO). Non-communicable diseases Country Profile 2018. Geneva: 2018 10.1515/pubhef-2016-0050

[3] World Health Organization, World Health Organisation. Global Status Report on Noncommunicable Diseases. Geneva: World Health Organization; 2014.

[4] Wu C-Y, Hu H-Y, Chou Y-J, Huang N, Chou Y-C, Li C-P. High Blood Pressure and All-Cause and Cardiovascular Disease Mortalities in Community-Dwelling Older Adults. Medicine (Baltimore) 2015;94:e2160 10.1097/MD.0000000000002160 26632749 PMC5059018

[5] Federal Ministry of Health. National Strategic Health Development Plan (NSHDP II). Abuja, Nigeria: 2018.

[6] Akinkugbe OO. Noncommunicable diseases in Nigeria: final report of a national survey. Lagos, Nigeria: 1997.

[7] Adeloye D, Basquill C, Aderemi A V., Thompson JY, Obi FA. An estimate of the prevalence of hypertension in Nigeria. J Hypertens 2015;33:230–42. 10.1097/HJH.0000000000000413 25380154

[8] Oguanobi Ejim ECE, Onwubere BJB Ike SOS, Anisiuba BBC Ikeh VVO, et al Pattern of cardiovascular disease amongst medical admissions in a regional teaching hospital in Southeastern Nigeria. Niger J Cardiol 2013;10:77–80. 10.4103/0189-7969.127005.

[9] Kolo PM, Jibrin YB, Sanya EO, Alkali M, Peter Kio IB, Moronkola RK. Hypertension-related admissions and outcome in a tertiary hospital in northeast Nigeria. Int J Hypertens 2012;2012:1–6. 10.1155/2012/960546. PMC3388339 22778913

[10] The World Bank. Population, total—Nigeria | Data 2020. https://data.worldbank.org/indicator/SP.POP.TOTL?locations=NG (accessed September 28, 2020).

[11] The World Bank. Life expectancy at birth, total (years)—Nigeria | Data 2020. https://data.worldbank.org/indicator/SP.DYN.LE00.IN?locations=NG (accessed September 28, 2020).

[12] Ekore RI, Ajayi IO, Arije A. Case finding for hypertension in young adult patients attending a missionary hospital in Nigeria. Afr Heal Sci 2009;9:193–9. 20589150 PMC2887025

[13] Bello B, Raji Y, Amira C, Braimoh R, Olowoyo O, Akodu B. Blood pressure pattern among adults in Lagos: Analysis of data from public health screenings. Niger J Gen Pract 2018;16:10 10.4103/NJGP.NJGP_20_17.

[14] Ogunmola OJ, Olaifa AO, Oladapo OO, Babatunde OA. Prevalence of cardiovascular risk factors among adults without obvious cardiovascular disease in a rural community in Ekiti State, Southwest Nigeria. BMC Cardiovasc Disord 2013;13:89 10.1186/1471-2261-13-89 24138186 PMC4016363

[15] Okpechi IG, Chukwuonye II, Tiffin N, Madukwe OO, Onyeonoro UU, Umeizudike TI, et al Blood Pressure Gradients and Cardiovascular Risk Factors in Urban and Rural Populations in Abia State South Eastern Nigeria Using the WHO STEPwise Approach. PLoS One 2013;8:e73403 10.1371/journal.pone.0073403 24039932 PMC3764162

[16] Oluyombo R, Olamoyegun MA, Olaifa O, Iwuala SO, Babatunde OA. Cardiovascular risk factors in semi-urban communities in southwest Nigeria: Patterns and prevalence. J Epidemiol Glob Health 2015;5:167–74. 10.1016/j.jegh.2014.07.002 25922326 PMC7320492

[17] Agaba EI, Akanbi MO, Agaba PA, Ocheke AN, Gimba ZM, Daniyam S, et al A survey of non-communicable diseases and their risk factors among university employees: a single institutional study. Cardiovasc J Afr 2017;28:377–84. 10.5830/CVJA-2017-021 28820539 PMC5885043

[18] Oguoma VM, Nwose EU, Skinner TC, Digban KA, Onyia IC, Richards RS. Prevalence of cardiovascular disease risk factors among a Nigerian adult population: relationship with income level and accessibility to CVD risks screening. BMC Public Health 2015;15:397 10.1186/s12889-015-1709-2 25925238 PMC4415344

[19] Government of Benue State Nigeria. Quick Facts–Government of Benue State 2020.

[20] National Agency for the Control of AIDS. NIGERIA PREVALENCE RATE–NACA Nigeria 2020.

[21] Ezeanolue EE, Obiefune MC, Ezeanolue CO, Ehiri JE, Osuji A, Ogidi AG, et al Effect of a congregation-based intervention on uptake of HIV testing and linkage to care in pregnant women in Nigeria (Baby Shower): A cluster randomised trial. Lancet Glob Heal 2015;3:e692–700. 10.1016/S2214-109X(15)00195-3 26475016 PMC5042903

[22] Ezeanolue EE, Obiefune MC, Yang W, Obaro SK, Ezeanolue CO, Ogedegbe GG. Comparative effectiveness of congregation- versus clinic-based approach to prevention of mother-to-child HIV transmission: Study protocol for a cluster randomized controlled trial. Implement Sci 2013;8 10.1186/1748-5908-8-62 23758933 PMC3700826

[23] Donato KA. Executive summary of the clinical guidelines on the identification, evaluation, and treatment of overweight and obesity in adults. Arch Intern Med 1998;158:1855–67. 10.1001/archinte.158.17.1855 9759681

[24] Chobanian A V., Bakris GL, Black HR, Cushman WC, Green LA, Izzo JL, et al The Seventh Report of the Joint National Committee on Prevention, Detection, Evaluation, and Treatment of High Blood Pressure: The JNC 7 Report. J Am Med Assoc 2003;289:2560–72. 10.1001/jama.289.19.2560. 12748199

[25] II U, CK I, BJ O, E A, O O, C O. High Prevalence and Low Awareness of Hypertension in a Market Population in Enugu, Nigeria Int J Hypertens 2011;2011 10.4061/2011/869675. PMC3038598 21331378

[26] Okpechi IG, Chukwuonye II, Tiffin N, Madukwe OO, Onyeonoro UU, Umeizudike TI, et al Blood Pressure Gradients and Cardiovascular Risk Factors in Urban and Rural Populations in Abia State South Eastern Nigeria Using the WHO STEPwise Approach. PLoS One 2013;8 10.1371/journal.pone.0073403 24039932 PMC3764162

[27] Oguoma VM, Nwose EU, Skinner TC, Digban KA, Onyia IC, Richards RS. Prevalence of cardiovascular disease risk factors among a Nigerian adult population: Relationship with income level and accessibility to CVD risks screening. BMC Public Health 2015;15 10.1186/s12889-015-1709-2 25925238 PMC4415344

[28] Isara AR, Okundia PO. The burden of hypertension and diabetes mellitus in rural communities in Southern Nigeria. Pan Afr Med J 2015;20 10.11604/pamj.2015.20.103.5619 26090051 PMC4458303

[29] Ezejimofor M, Uthman O, Chen YF, Ezejimofor B, Ezeabasili A, Stranges S, et al Magnitude and pattern of hypertension in the Niger Delta: A systematic review and meta-analysis of community-based studies. J Glob Health 2018;8 10.7189/jogh.08.010420 29899980 PMC5997369

[30] TM D, OF Y, AH A, GI O, SN C. Cardiovascular Risk Factors in a Suburban Community in Nigeria. Int J Hypertens 2018;2018 10.1155/2018/6898527. PMC5899850 29805795

[31] Buford TW. Hypertension and aging. Ageing Res Rev 2016;26:96–111. 10.1016/j.arr.2016.01.007 26835847 PMC4768730

[32] Okpechi IG, Chukwuonye II, Tiffin N, Madukwe OO, Onyeonoro UU, Umeizudike TI, et al Blood Pressure Gradients and Cardiovascular Risk Factors in Urban and Rural Populations in Abia State South Eastern Nigeria Using the WHO STEPwise Approach. PLoS One 2013;8:e73403 10.1371/journal.pone.0073403 24039932 PMC3764162

[33] Ezeala-Adikaibe BA, Orjioke C, Ekenze OS, Ijoma U, Onodugo O, Okudo G, et al Population-based prevalence of high blood pressure among adults in an urban slum in Enugu, South East Nigeria. J Hum Hypertens 2016;30:285–91. 10.1038/jhh.2015.49 26016595

[34] Ekwunife OI, Udeogaranya PO, Nwatu IL. Prevalence, awareness, treatment and control of hypertension in a nigerian population. Health (Irvine Calif) 2010;02:731–5. 10.4236/health.2010.27111.

[35] Isezuo SA, Sabir AA, Ohwovorilole AE, Fasanmade OA. Prevalence, associated factors and relationship between prehypertension and hypertension: A study of two ethnic African populations in Northern Nigeria. J Hum Hypertens 2011;25:224–30. 10.1038/jhh.2010.56 20555358

[36] Chukwuma A, Gong E, Latypova M, Fraser-Hurt N. Challenges and opportunities in the continuity of care for hypertension: A mixed-methods study embedded in a primary health care intervention in Tajikistan. BMC Health Serv Res 2019;19:1–13. 10.1186/s12913-019-4779-5 31796016 PMC6889695

[37] Awuah RB, Anarfi JK, Agyemang C, Ogedegbe G, Aikins A de-G. Prevalence, awareness, treatment and control of hypertension in urban poor communities in Accra, Ghana. J Hypertens 2014;32:1203–10. 10.1097/HJH.0000000000000165 24721931

[38] Geldsetzer P, Manne-Goehler J, Marcus ME, Ebert C, Zhumadilov Z, Wesseh CS, et al The state of hypertension care in 44 low-income and middle-income countries: a cross-sectional study of nationally representative individual-level data from 1·1 million adults. Lancet 2019;394:652–62. 10.1016/S0140-6736(19)30955-9 31327566

[39] Lu J, Lu Y, Wang X, Li X, Linderman GC, Wu C, et al Prevalence, awareness, treatment, and control of hypertension in China: data from 1·7 million adults in a population-based screening study (China PEACE Million Persons Project). Lancet 2017;390:2549–58. 10.1016/S0140-6736(17)32478-9 29102084

[40] Wall HK, Hannan JA, Wright JS. Patients with undiagnosed hypertension hiding in plain sight. JAMA—J Am Med Assoc 2014;312:1973–4. 10.1001/jama.2014.15388 25399269 PMC4596255

[41] Chow CK, Teo KK, Rangarajan S, Islam S, Gupta R, Avezum A, et al Prevalence, awareness, treatment, and control of hypertension in rural and urban communities in high-, middle-, and low-income countries. JAMA—J Am Med Assoc 2013;310:959–68. 10.1001/jama.2013.184182. 24002282

[42] Ahmed S, Tariqujjaman M, Rahman MA, Hasan MZ, Hasan MM. Inequalities in the prevalence of undiagnosed hypertension among Bangladeshi adults: Evidence from a nationwide survey. Int J Equity Health 2019;18 10.1186/s12939-019-0930-5 30770739 PMC6377713

[43] Geldsetzer P, Manne-Goehler J, Marcus M-E, Ebert C, Zhumadilov Z, Wesseh CS, et al The state of hypertension care in 44 low-income and middle-income countries: a cross-sectional study of nationally representative individual-level data from 1·1 million adults. Lancet 2019;394:652–62. 10.1016/S0140-6736(19)30955-9 31327566

[44] Kayima J, Wanyenze RK, Katamba A, Leontsini E, Nuwaha F. Hypertension awareness, treatment and control in Africa: A systematic review. BMC Cardiovasc Disord 2013;13:54 10.1186/1471-2261-13-54 23915151 PMC3750220

[45] Oguanobi N, Onwubere B, Aneke E, Anisiuba B, Ejim E, Ike S, et al Pattern of cardiovascular disease amongst medical admissions in a regional teaching hospital in Southeastern Nigeria. Niger J Cardiol 2013;10:77 10.4103/0189-7969.127005.

[46] Kolo PM, Jibrin YB, Sanya EO, Alkali M, Peter Kio IB, Moronkola RK. Hypertension-related admissions and outcome in a tertiary hospital in northeast Nigeria. Int J Hypertens 2012;2012 10.1155/2012/960546. PMC3388339 22778913

[47] Ezeala-Adikaibe B, Aneke E, Aneke C, Ezeala-Adikaibe N, Mbadiwe M, Chime P, et al Pattern of medical admissions at Enugu State University of Science and Technology Teaching Hospital: A 5 year review. Ann Med Health Sci Res 2014;4:426 10.4103/2141-9248.133472 24971220 PMC4071745

[48] Adeoti AO, Ajayi EA, Ajayi AO, Dada SA, Fadare JO, Akolawole M, et al Pattern and Outcome of Medical Admissions in Ekiti State University Teaching Hospital, Ado-Ekiti- A 5 Year Review. Am J Med Med Sci 2015;5:92–8. 10.1006/jmre.2000.2080.

[49] Oladapo OO, Salako L, Sadiq L, Shoyinka K, Adedapo K, Falase AO. Target-organ damage and cardiovascular complications in hypertensive Nigerian Yoruba adults: A cross-sectional study. Cardiovasc J Afr 2012;23:379–84. 10.5830/CVJA-2012-021 22914995 PMC3721802

[50] Gbadegesin A, Okunola O, Ayodele O, Arogundade F, Sanusi A, Akinsola A. Renal risk profiling in newly diagnosed hypertensives in an urban population in Nigeria. Afr Health Sci 2019;19:2863–73. 10.4314/ahs.v19i4.8 32127862 PMC7040353

[51] Adedini SA, Odimegwu C, Bamiwuye O, Fadeyibi O, De Wet N. Barriers to accessing health care in Nigeria: Implications for child survival. Glob Health Action 2014;7:1–10. 10.3402/gha.v7.23499 24647128 PMC3957799

[52] Ofoli JNT, Ashau-Oladipo T, Hati SS, Ati L, Ede V. Preventive healthcare uptake in private hospitals in Nigeria: A cross-sectional survey (Nisa premier hospital). BMC Health Serv Res 2020;20:273 10.1186/s12913-020-05117-5 32238153 PMC7114808

[53] Okubadejo NU, Ozoh OB, Ojo OO, Akinkugbe AO, Odeniyi IA, Adegoke O, et al Prevalence of hypertension and blood pressure profile amongst urban-dwelling adults in Nigeria: a comparative analysis based on recent guideline recommendations. Clin Hypertens 2019;25 10.1186/s40885-019-0112-1 31016027 PMC6463661

[54] Prevalence and correlates of prehypertension and hypertension among adults in Delta State, Nigeria: a cross-sectional community-based study | Ghana Medical Journal n.d. https://www.ajol.info/index.php/gmj/article/view/194537 (accessed May 20, 2020). 10.4314/gmj.v54i1.8 PMC7445701 32863413

[55] Okwuonu C, Ngoka S, Uwanurochi K, Mbanaso A, Chimezie O, Eze T. Towards prevention of hypertension in Nigeria: A study of prehypertension and its associations among apparently healthy adults in Umuahia, South-East Nigeria. Int J Prev Med 2015;6:61 10.4103/2008-7802.160968 26288705 PMC4521306

[56] Kanegae H, Oikawa T, Kario K. Should Pre-hypertension Be Treated? Curr Hypertens Rep 2017;19:1–9. 10.1007/s11906-017-0789-z 29046988

[57] Egan BM, Stevens-Fabry S. Prehypertension—Prevalence, health risks, and management strategies. Nat Rev Cardiol 2015;12:289–300. 10.1038/nrcardio.2015.17 25687779

[58] Mecha JO, Kubo EN, Odhiambo CO, Kinoti FG, Njau K, Yonga G, et al Burden of prehypertension among adults in Kenya: A retrospective analysis of findings from the Healthy Heart Africa (HHA) Programme. BMC Public Health 2020;20:281 10.1186/s12889-020-8363-z 32126994 PMC7055018

[59] Akpa OM, Made F, Ojo A, Ovbiagele B, Adu D, Motala AA, et al Regional patterns and association between obesity and hypertension in Africa: Evidence from the H3Africa Chair study. Hypertension 2020;75:1167–78. 10.1161/HYPERTENSIONAHA.119.14147 32172619 PMC7176339

[60] Puddey IB, Mori TA, Barden AE, Beilin LJ. Alcohol and Hypertension—New Insights and Lingering Controversies. Curr Hypertens Rep 2019;21 10.1007/s11906-019-0984-1 31494743

[61] Roerecke M, Tobe SW, Kaczorowski J, Bacon SL, Vafaei A, Hasan OSM, et al Sex-specific associations between alcohol consumption and incidence of hypertension: A systematic review and meta-analysis of cohort studies. J Am Heart Assoc 2018;7 10.1161/JAHA.117.008202 29950485 PMC6064910

[62] Oguoma VM, Nwose EU, Skinner TC, Richards RS, Bwititi PT. Diet and lifestyle habits: Association with cardiovascular disease indices in a Nigerian sub-population. Diabetes Metab Syndr Clin Res Rev 2018;12:653–9. 10.1016/j.dsx.2018.04.007. 29673925

[63] Olawuyi AT, Adeoye IA. The prevalence and associated factors of noncommunicable disease risk factors among civil servants in Ibadan, Nigeria. PLoS One 2018;13 10.1371/journal.pone.0203587 30212508 PMC6136760

[64] Adeloye D, Olawole-Isaac A, Auta A, Dewan MT, Omoyele C, Ezeigwe N, et al Epidemiology of harmful use of alcohol in Nigeria: a systematic review and meta-analysis. Am J Drug Alcohol Abuse 2019;45:438–50. 10.1080/00952990.2019.1628244 31246100

[65] Pew Research Center. Tolerance and Tension: Islam and Christianity in Sub-Saharan Africa | Pew Research Center n.d. https://www.pewforum.org/2010/04/15/executive-summary-islam-and-christianity-in-sub-saharan-africa/ (accessed October 4, 2020).

